# A decline in PABPN1 induces progressive muscle weakness in Oculopharyngeal muscle dystrophy and in muscle aging

**DOI:** 10.18632/aging.100567

**Published:** 2013-06-16

**Authors:** Seyed Yahya Anvar, Yotam Raz, Nisha Verwey, Barbara van der Sluijs, Andrea Venema, Jelle J Goeman, John Vissing, Silvère M van der Maarel, Peter A.C. ‘t Hoen, Baziel G.M. van Engelen, Vered Raz

**Affiliations:** ^1^ Center for Human and Clinical Genetics, Leiden University Medical Center, the Netherlands; ^2^ Department of Gerontology and Geriatrics, Leiden University Medical Center, the Netherlands; ^3^ Department of Medical Statistics and Bioinformatics, Leiden University Medical Center, the Netherlands; ^4^ Department of Neurology, Radboud University Nijmegen Medical Center, the Netherlands; ^5^ Department of Anaesthesia, Canisius-Wilhelmina Hospital, Nijmegen, the Netherlands; ^6^ Neuromuscular Research Unit and Department of Neurology, Rigshospitalet Copenhagen, Denmark

**Keywords:** Type 2 diabetes, TCF7L2, centenarians, extreme phenotypes, age-related diseases

## Abstract

Oculopharyngeal muscular dystrophy (OPMD) is caused by trinucleotide repeat expansion mutations in Poly(A) binding protein 1 (PABPN1). PABPN1 is a regulator of mRNA stability and is ubiquitously expressed. Here we investigated how symptoms in OPMD initiate only at midlife and why a subset of skeletal muscles is predominantly affected. Genome-wide RNA expression profiles from *Vastus lateralis* muscles human carriers of expanded-PABPN1 at pre-symptomatic and symptomatic stages were compared with healthy controls. Major expression changes were found to be associated with age rather than with expression of expanded-PABPN1, instead transcriptomes of OPMD and elderly muscles were significantly similar (P<0.05). Using k-means clustering we identified age-dependent trends in both OPMD and controls, but trends were often accelerated in OPMD. We report an age-regulated decline in PABPN1 levels in *Vastus lateralis* muscles from the fifth decade. In concurrence with severe muscle degeneration in OPMD, the decline in PABPN1 accelerated in OPMD and was specific to skeletal muscles. Reduced PABPN1 levels (30% to 60%) in muscle cells induced myogenic defects and morphological signatures of cellular aging in proportion to PABPN1 expression levels. We suggest that PABPN1 levels regulate muscle cell aging and OPMD represents an accelerated muscle aging disorder.

## INTRODUCTION

Protein aggregation is a pathological hallmark in a large spectrum of late-onset neurodegenerative disorders. Single amino acid repeat expansions are among the common genetic cause of those disorders [1]. These disorders are recognized by the presence of salt-resistant insoluble bodies, resulting from accumulation of the expanded mutant proteins. Accumulation of aggregated proteins is associated with impaired protein homeostasis and the ubiquitin proteasome system (UPS), which advances protein aggregation [2]. The presence of disease-associated protein aggregation is often an indication of failure of cellular processes maintaining protein homeostasis [3]. Nevertheless, despite their evident occurrence in late-onset neurodegenerative disorders, a pathogenic role of these aggregates remains controversial [4].

Most of the expansion mutations create elongated polyglutamine (poly-Q) tracts, but a subset of diseases is caused by polyalanine tract expansions [5]. Autosomal dominant oculopharyngeal muscular dystrophy (OPMD) is caused by a poly-alanine repeat expansion mutation in the gene encoding for *Poly(A) Binding Protein Nuclear 1* (*PABPN1*) [6]. OPMD is characterized by progressive ptosis, dysphagia, and proximal limb muscle weakness that typically appear from the fifth decade [5]. Accumulation of expanded (exp)PABPN1 into insoluble intranuclear inclusions in skeletal muscles is the pathological hallmark of OPMD [7, 8]. Because of the many commonalities in the protein aggregation process, it is often considered a paradigm for the neurodegenerative protein aggregation disorders with the unique advantage of having access to pre-symptomatic and symptomatic tissue. Naturally occurring inclusions of wild-type (WT) PABPN1 have also been reported [9]. In contrast to aggregates of expPABPN1, those of the WT protein are not associated with disease. In muscle cell models both WT and expPABPN1 form nuclear inclusions, but expPABPN1 is more prone to aggregation [10]. This increased aggregation of expPABPN1 can be explained, in part, by its reduced poly-ubiquitination and slower protein turnover compared with WT PABPN1 [10].

The impact of aggregated PABPN1 on cellular defects is not fully understood. In high overexpression situations expPABPN1, but not WT PABNP1, induces cell death [11, 12]. Therefore, a toxic gain-of-function was suggested for mutant PABPN1 (reviewed in: [5]). In cellular models with low overexpression levels that are similar to levels of endogenous PABPN1, and therefore mimic better the situation in heterozygous patients, apoptosis was not observed [10, 13]. In myotube cultures with low PABPN1 overexpression, deregulation of the ubiquitin proteasome system (UPS) was the most affected cellular pathway [10]. Deregulation of the UPS was also found to be most prominent in a cross-species study for OPMD [14], and reduced proteasome activity was found in the A17.1 mouse model for OPMD [15]. This suggests that disruption of cellular processes regulating protein homeostasis, rather than cell death, could cause muscle degeneration in OPMD.

High overexpression of expPABPN1 or depletion of PABPN1 by RNA interference in muscle causes myogenic defects [16-20]. These models have been instrumental to demonstrate that manipulations in PABPN1 expression levels cause muscle symptoms. As yet, however, it is unclear how ubiquitously expressed PABPN1 causes predominant skeletal muscle pathology and why symptoms in OPMD initiate only after midlife. Here we investigated the hypothesis that OPMD represents accelerated aging myopathy. We compared genome-wide expression profiles from *Vastus lateralis* (*VL*) muscles of OPMD patients and elderly and identified significant similarities. In concurrence with severe muscle weakness in OPMD, age-dependent molecular changes are often augmented in OPMD muscles. We also identified an age-dependent decline in PABPN1 levels. This decrease in PABPN1 levels starts only from the fifth decade and is accelerated in OPMD. In culture, PABPN1 down-regulation induces muscle cell aging and myogenesis defects that are proportional to a decrease in PABPN1 levels. We suggest that PABPN1 regulates muscle cell aging.

## RESULTS

### Transcriptome changes in OPMD are associated with initiation of symptoms

Symptoms in OPMD often initiate with eye lid muscle weakness (ptosis) or and swallowing difficulties (dysphagia) [7], but muscle weakness in upper limbs was also reported [21]. In our patient cohort limb muscle weakness was often highly abundant (Table 1). Muscle strength in 33 genetically confirmed OPMD patients (Supplementary Table 1) was determined with the *Medical Research Council* (MRC) scale, where MRC <5 represents clinical symptoms. An association between muscle strength and age was statistically assessed from 11 muscle groups, from which significant association was found in 8 muscle groups (Table 1). Using the same muscle strength measures, in carriers of expPABPN1 under 42 years-old muscle weakness was not observed (Supplementary Table 1). This suggests that age is major confounder in muscle weakness in OPMD.

**Table 1:** Muscle weakness in OPMD is age-dependent

Muscle (group)	Beta of MRC age-associated ^1^	*p-value*
Iliopsoas	−0.07 (0.02)	**3.8E-04**
Biceps	−0.03 (0.01)	**2.0E-03**
Hamstrings	−0.04 (0.01)	**5.0E-03**
Feet	−0.02 (0.01)	**5.0E-03**
Neck extensors	−0.03 (0.01)	**6.0E-03**
Med. Gluteal	−0.05 (0.02)	**2.0E-02**
Quadriceps	−0.02 (0.01)	**3.0E-02**
Triceps	−0.01 (0.01)	**4.8E-02**
Deltoid	−0.03 (0.01)	8.0E-02
Hip extensors	−0.08 (0.02)	2.7E-01
Neck flexors	−0.01 (0.02)	4.5E-01

Regression analysis of MRC scores dependent on age (in years) of patients at examination. Betas (standard errors) are provided. P-values are adjusted for gender. N= 33; Mean age in years: 60.4 (± 11.3); Females 49%.

In OPMD *VL* muscles we identified genome-wide changes in gene expression [14]. Here we assessed whether these changes in OPMD are associated with with the expression of expPABPN1 and/or with age. We first compared the transcriptome of OPMD patients with that of expPABPN1 carriers at a pre-symptomatic stage. Expression profiles for each group were generated after subtraction to an age-matched control group: for the pre-symptomatic group mean age was 36 ± 3.5 N=6 and 9 (carriers and controls, respectively) and in the symptomatic group mean age was 56 ± 5 years N=9 and 13 (carriers and controls, respectively) (Supplementary Table 1). The genes found to be affected in OPMD (N=1572 genes; *p-value* < 0.01) were clustered into 73 functional Kyoto Encyclopaedia of Genes and Genomes (KEGG) pathways and were highly similar to those identified in OPMD animal models (Supplementary Table 2). With the same p-value cut-off only 473 genes were identified in the pre-symptomatic group and those did not enrich in any KEGG pathway. This suggests that gene deregulation in pre-symptomatic have little functional impact. After applying the false discovery rate (FDR)-correction, 149 genes were found to be deregulated in the symptomatic group while in the pre-symptomatic group no gene passed those stringent criteria. This analysis thus shows that in OPMD muscle transcriptome changes are associated with symptoms, while changes caused by the presence of expPABPN1 are only minor with limited functional impact. Although more confidence of gene deregulation is obtained after FDR correction, since our dataset is relatively small we selected the deregulated genes with *p*-value 0.01 for further analysis.

### Expression profiles in OPMD muscles and in muscles of elderly are highly similar

We first investigated the possibility of molecular similarities between OPMD muscle and muscle aging using functional groups. The affected KEGG pathways in OPMD were highly similar to those found in an independent study of normal muscle aging [22]. From the 75 KEGG pathways that are significantly affected in this muscle aging study only 3 (96% similarity) were not identified in OPMD (Supplementary Table 2).

**Figure 1:**
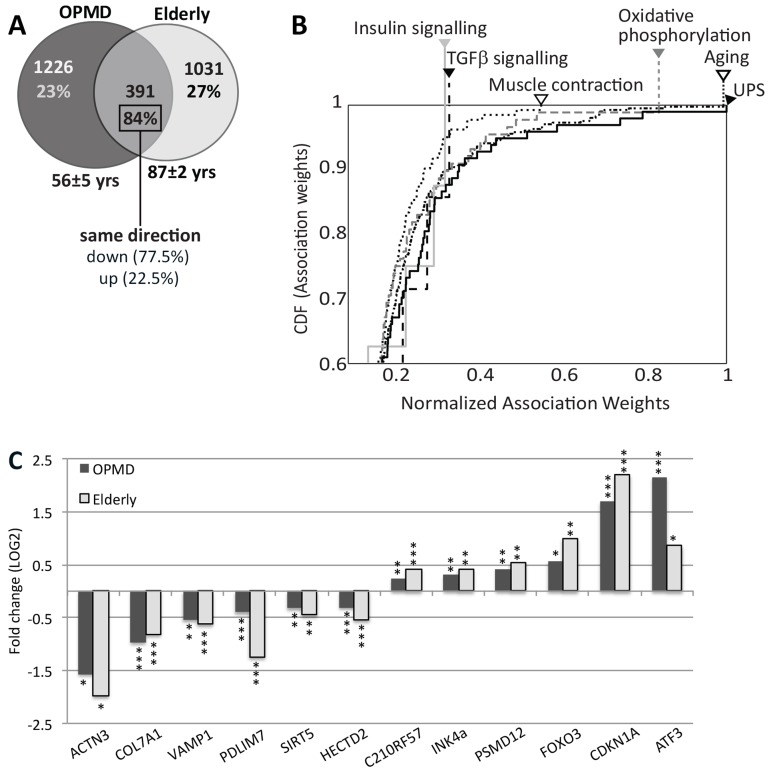
Similar expression profiles in OPMD and elderly muscles (**A**) Venn diagram shows the number of affected genes (p ≤0.01) in OPMD, elderly overlapping genes. The percentage of similar gene direction and the direction (up or down) are listed. For each group the mean age ± standard deviation are denoted. (**B**) Cumulative distribution function (CDF) plots show the distribution of normalized literature association-weights of commonly deregulated genes between OPMD and elderly with the concepts: Aging, Muscle contraction, Oxidative phospho-rylation, insulin signalling, TGFβsignalling, and the ubiquitin-proteasome system (UPS). Arrowheads indicate the maximum association weights. The further the curve shifts to the right, the higher the associations of the affected genes with the indicated concepts are. (**C**) Bar-chart shows fold-change of selected known age-regulated genes in elderly and in OPMD. *P-values* are indicated: * *P*≤0.01, ** *P*<0.005, *** *P*<0.0005.

If molecular changes in OPMD are similar to these in muscle aging similarity in dysregulated genes and expression trends is expected. We compared OPMD expression profiles (mean age 56 ± 5) with those of elderly (>85 years) (Supplementary Table 1). It is relevant to note that OPMD patients were under 70 years-old and therefore may not considered as elderly. *VL* muscles from healthy controls were sampled same as the OPMD biopsies and expression profiles were generated on the same platform. Expression profiles in OPMD and elderly were generated with the same control group (mean age 56 ± 5). Close to one third of the genes affected in elderly overlapped with those affected in OPMD (Figure 1A), and the same directionality in fold-change was found for 84% of these genes. The majority of the overlapping genes (77%) were down regulated (Figure 1A). This observation is consistent with other studies that found a prominent transcriptional down-regulation in neurons from the elderly [23]. Gene Ontology (GO) analysis of OPMD- or elderly- regulated genes also revealed high similarities between significantly affected cellular and molecular functions (Table 2).

**Table 2:** Top 10 affected GO-terms in OPMD and elderly

OPMD			Elderly		
GO term	*P-value*	Nr	GO Term	*P-value*	Nr
0005681~spliceosome	1.1E-07	29	0005681~spliceosome	5.9E-05	22
0005581~collagen	0.002	9	0044429~mitochondrial part	1.0E-04	61
0044420~extracellular matrix part	0.003	18	0006511~ubiquitin-dependent protein catabolic process	0.006	27
0006511~ubiquitin-dependent protein catabolic process	0.004	31	0043292~contractile fiber	0.008	16
0006281~DNA repair	0.004	35	0015629~actin cytoskeleton	0.009	28
0000123~histone acetyltransferase complex	0.007	10	0004311~farnesyltranstrans-ferase activity	0.023	3
0016570~histone modification	0.019	17	0033279~ribosomal subunit	0.027	15
0006915~apoptosis	0.026	59	0006006~glucose metabolic process	0.032	17
0006405~RNA export from nucleus	0.031	8	0003678~DNA helicase activity	0.040	7
0044429~mitochondrial part	0.044	54	0043065~positive regulation of apoptosis	0.051	37

Enriched GO terms were identified with the list of OPMD- or Elderly-affected genes (P-value ?0.01). The numbers (Nr) of genes mapped to each GO-term are indicated.

We then investigated whether aging- or OPMD-regulated genes are correspondingly enriched in recognized aging concepts. We employed the literature association method [24] with the concepts: aging, muscle contraction, oxidative phosphorylation, insulin signalling, tumour growth factor (TGFβ) signalling and the ubiquitin proteasome system (UPS). The literature association of every gene with each of the 6 concepts was determined and normalized association-weights were assigned. Association enrichment for the significantly regulated genes in each concept was evaluated with the Cumulative Distribution Function (CDF). The distribution of association-weights for the aging concept had a maximum score indicating that the common genes between OPMD and elderly indeed represent well-known aging genes (Figure 1B). As expected, significant scores were also found for oxidative phosphorylation and muscle contraction. Next to the aging concept, the UPS also rated with a maximum score, suggesting that UPS genes have highest impact for both OPMD and elderly. The high significant score indicates that the association is not random (*P* = 4.3×10^−39^, 8.1×10^−25^ and 2.4×10^−26^, for UPS, oxidative phosphorylation and muscle contraction respectively). In contrast, the distributions of weight-associations for genes in the insulin or TGF-β signalling pathways were not strong (Figure 1B), and *P*-values did not differ from a theoretical random distribution. This analysis suggests that similar genes involved in the UPS, mitochondria and muscle contraction affect muscle weakness in both elderly and in OPMD. The genes affecting the insulin or TGF-β signalling pathways, however, differ between OPMD and elderly. To visualize the molecular similarity between OPMD and elderly we plotted the mean fold change for well-known aging-regulated genes [25] (Figure 1C). Together our analysis revealed a significant consistencyin molecular signatures that characterize *VL* muscles in OPMD patients and in elderly.

### Transcriptome of an OPMD mouse model clusters with muscle aging transcriptome

**Figure 2:**
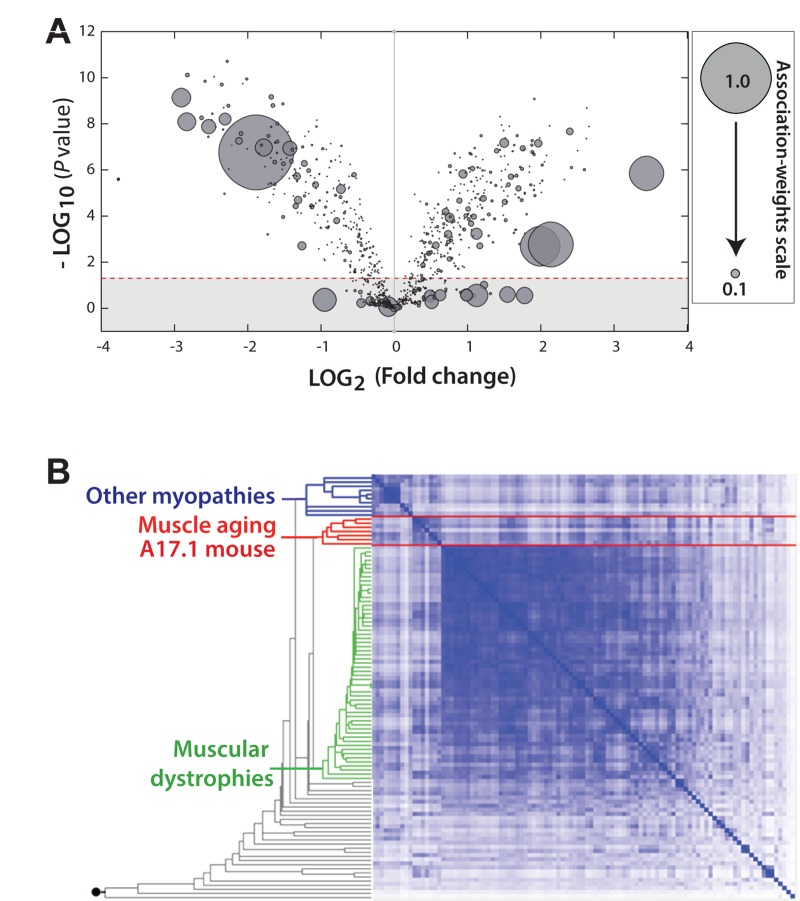
The transcriptome of the OPMD mouse model is highly associated with aging (**A**) Volcano plot shows the distribution of significantly deregulated genes (*P* = 0.05; indicated with a dashed line) in 6 week-old A17.1 mice against fold-change. Smaller *P*-value and higher fold-change suggests a higher impact in OPMD. For every gene a literature-associated weight with the ‘*Aging'* concept is assigned, and a normalized association-weight is presented with a circle on a linear scale between 0.05 and 1, where 1 equals the highest association. (**B**) Hierarchical clustering arrangements of 104 datasets in a literature-aided meta-analysis. Shades of blue indicate degree of similarities: from weak (white) to strong (dark blue). Three skeletal muscle aging-related datasets cluster together with the OPMD dataset of 6 week-old mice (highlighted in red). The clusters associated with muscular dystrophies and other myopathies are highlighted in green and blue, respectively.

We further studied the association of the OPMD transcriptome with aging using the transcriptome of the A17.1 mouse at 6-weeks old, as muscle weakness is not obvious at that age (Trollet et al., 2010). In the A17.1 mouse expPABPN1 is overexpressed under a muscle-specific promoter resulting in severe muscle weakness (Davies et al., 2005) and muscle atrophy at an early age [15]. A literature-aided association study of the differentially expressed genes in the A17.1 muscles of 6 weeks-old mice showed that a large subset of these genes strongly associates with the a*ging concept* (Figure 2A). The fold-change for the genes with a high association-weight score was large (Figure 2A), indicating that well-known aging genes are highly dysregulated in this mouse model.

For an unbiased approach we performed an unsupervised meta-analysis with 104 microarray studies of muscle development and muscle disorders [26], including the transcriptome from muscle of 6-weeks old A17.1 mice. With this analysis similar transcriptional changes were grouped into three main clusters (Figure 2B). The transcriptome of the 6 week-old A17.1 mouse strictly grouped together with those related to skeletal muscle aging [22, 27], but not with datasets from other muscular dystrophies or myopathies (Figure 2B). This suggests that the high overexpression of expPABPN1 in the OPMD mouse model could induce similar molecular changes as in normal muscle aging.

### Expression trends are accelerated in OPMD

**Figure 3:**
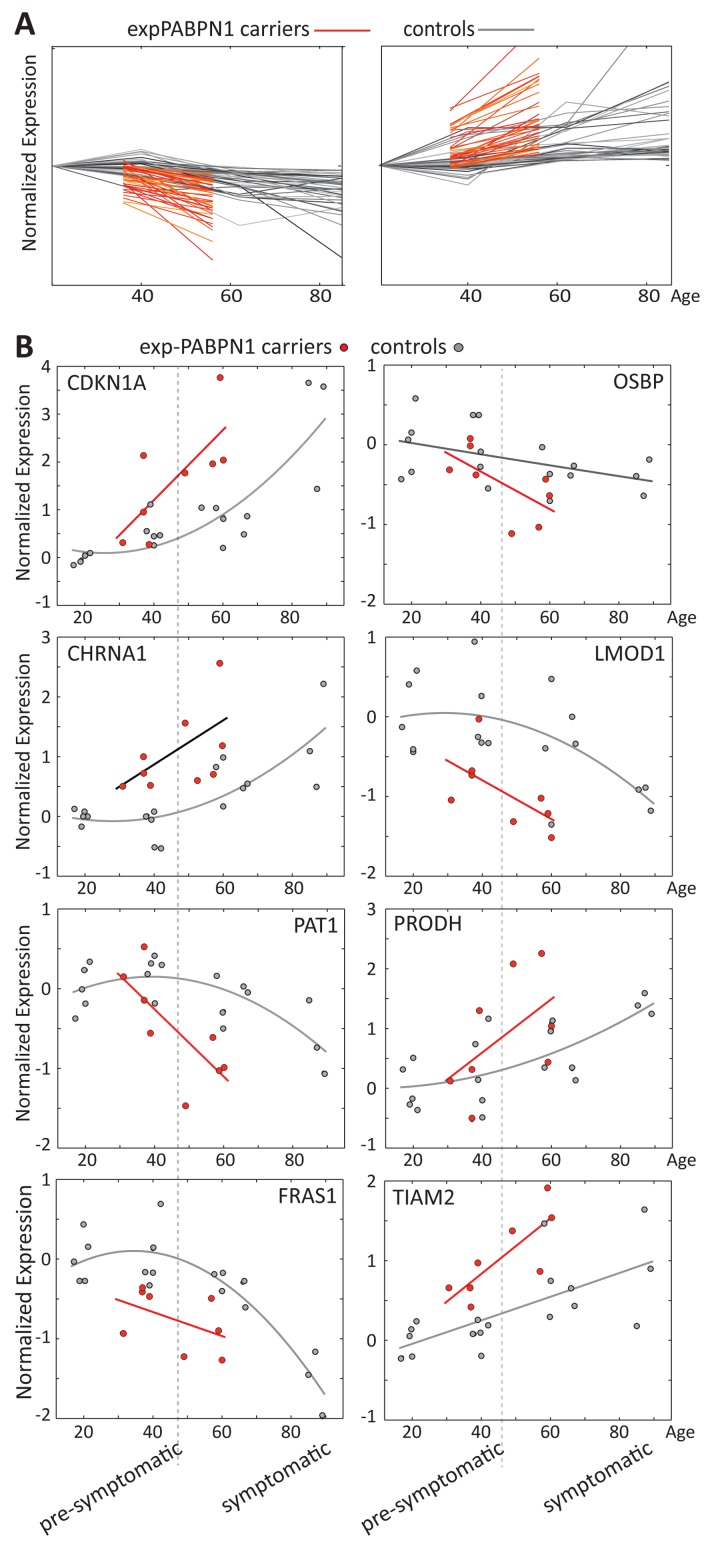
Age regulated gene expression trends change faster in OPMD (**A**) Expression trends in controls (grey) and expPABPN1 carriers (red). Similar expression trends were identified with k-means clustering using probe ID. (**B**) Examples of expression trends of 8 genes from clusters in **A**, in healthy controls (grey) or in exPABPN1 carriers at pre-symptomatic and symptomatic stages (red). Pre-symptomatic and symptomatic stages of OPMD are indicated.

Close to 30 years separate the OPMD patients in our study from the elderly, but the remarkable overlap in expression profiles suggests that changes in OPMD initiate earlier or progress faster compared with normal aging. To identify expression trends k-means clustering was applied on the overlapping probes between OPMD and elderly. We compared trends per probe in a dataset derived from the healthy cohort age 17-89 years to those derived from expPABPN1 carriers age 31-74, and subsequently, unique genes were identified using Entrez ID. Two clusters of significant age-regulated genes showing accelerated (N=35) or decelerated (N=39) trends in OPMD were identified (Figure 3A, Supplementary Table 3). Examples of trends for selected genes are presented in Figure 3B. Among those are well-known regulators of aging such as the cell cycle regulators *CDKN1A* (p21) and OSBP, regulators of muscle cells and energy metabolism like *LMOD1* and *CHRNA1* or *FAT1* and *PRODH*. These genes with accelerated expression changes in OPMD could contribute to disease progression and muscle weakness.

### *PABPN1* levels significantly decreases with age and in OPMD

**Figure 4:**
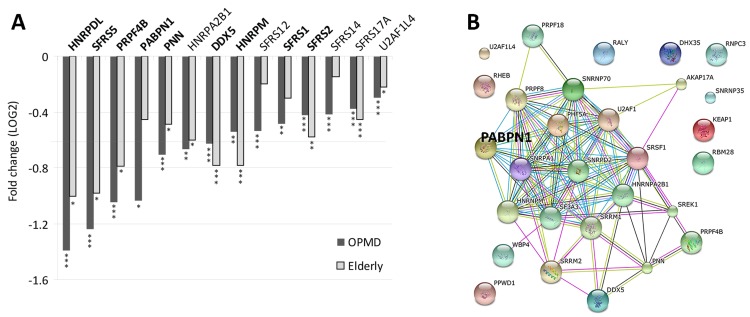
Deregulation of the Spliceosome in both OPMD and elderly (**A**) Bar-chart shows fold-change of spliceosome genes in OPMD and elderly from the transcriptome studies. *P-values* are indicated: * *P*<0.05, ** *P*<0.005, *** *P*<0.0005. In bold are gene-hubs in the OPMD-affected spliceosome network (**B**). A schematic gene network presentation of OPMD-regulated genes that are grouped in the spliceosome GO category. PABPN1 is highlighted

The mRNA processing and spliceosome GO-terms were the most significantly affected in both OPMD and elderly datasets (Table 2). Noticeably, the majority of the dysregulated genes in this category were down regulated (Figure 4A). Network analysis suggests that central hubs are affected in both OPMD and elderly, including PABPN1 (Figure 4B).

**Figure 5:**
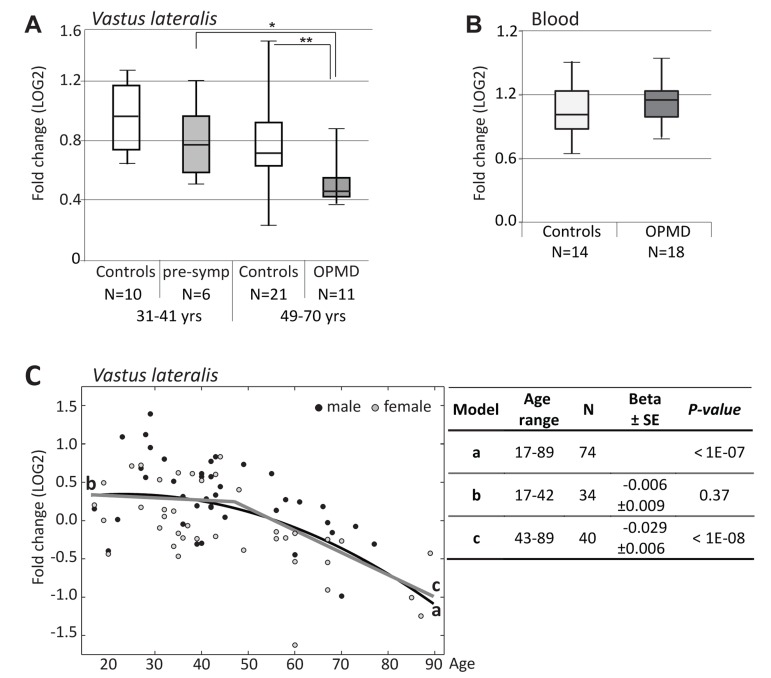
RT-qPCR analysis of PABPN1 expression trends in OPMD and during muscle aging (**A**) Box plot shows *PABPN1*expression in *Vastus lateralis* from expPABPN1 carriers at a pre-symptomatic (pre-symp) or symptomatic (OPMD) stages, and age-matching control groups. (**B**) Box plot shows*PABPN1*expression in blood form OPMD patients and controls. (**C**) Scatter plot shows *PABPN1*expression in 78 healthy controls age 17-89 years. Male and female samples are indicated in black and grey, respectively. A quadratic fit is shown with a black line (a), and linear fits are for the age groups: 17-42 years (b) or 43-89 years (c) are denoted in grey. The table summarizes p-values and Beta ± standard errors, which were calculated after gender correction. Fold-changes were calculated after normalization of *GUSB* housekeeping gene and to control group age 17-22 years (**A, C**) or age matching controls (B). N denotes the number of samples in each group.

Since mutations in *PABPN1* are the genetic cause for OPMD, we next validated the decline in *PABPN1* mRNA levels using RT-qPCR using an extended cohort of skeletal muscle biopsies. A significant and pronounced decrease in expression was found in OPMD compared with age-matching controls, while at the pre-symptomatic stage PABPN1 levels did not significantly change (Figure 5A). This suggests that symptoms in OPMD patients are associated with a decrease in *PABPN1* expression.

Next, levels of *PABPN1* mRNA during aging were determined in *VL* from 74 healthy controls age 17-89 using RT-qPCR. Fold-change was calculated from *GUSB* housekeeping gene and young control group at age 17-22 years. A significant decrease in *PABPN1* expression with age was observed with a quadratic model (Figure 5C). Using two sequential linear models the decline in this dataset was found from 43 years onwards, whereas between 17-42 years the change in expression levels was insignificant (Figure 5C). Together, our analysis reveals that PABPN1 expression declines with aging from the fourth decade onwards and that this decline is accelerated in OPMD.

PABPN1 is expressed in every cell whilst symptoms in OPMD are predominantly restricted to a subset of skeletal muscles. To investigate whether the decrease in *PABPN1* expression can explain the tissue specificity in OPMD, *PABPN1* levels in blood samples of OPMD patients and controls was determined using RT-qPCR. *PABPN1* levels in blood were unchanged between OPMD patients and age-matching controls (Figure 5B), indicating that the decrease in *PABPN1* expression in OPMD is more prominent in skeletal muscles. An age-associated decrease in *PABPN1* levels was also not found in cross-sectional microarray studies from blood, parotid glands, kidney cortex and medulla (Table 3). In frontal brain cortex we identified a slight decrease in *PABPN1* expression (Table 3), which started only after the age of 70 years (Table 3). Also in *Rectus abdominis* muscles, where muscle aging is not so prominent [28], *PABPN1* expression did not change with age (Table 3). Together, this suggests that the age-associated decrease in PABPN1 expression marks aging of human skeletal muscles that are typically affected in OPMD.

**Table 3:** Changes in PABPN1 expression depends on chronological age are muscle specific

Tissue	Age	Beta	*P-value*
***Vastus lateralis*** muscles	17-42 y (N = 41) **43-89 y (N = 34)**	−0.006 (0.009) **-0.029 (0.006)**	0.37 **<0.0001**
**Frontal Brain Cortex**	26-69 y (N = 17) **70-95 y (N = 13)**	0.002 (0.007) **-0.018 (0.008)**	0.73 **0.04**
**Blood**	42 – 102 y (N = 150)	0.001 (0.003)	0.69
**Kidney Cortex**	27 – 92 y (N = 72)	−0.001 (0.002) -0.001 (0.002) -0.003 (0.002)	0.76 0.42 0.15
**Kidney Medulla**	29 – 92 y (N = 61)	−0.003 (0.002) 0.001 (0.002) -0.004 (0.002)	0.11 0.76 0.06
**Rectus *Abdominis***	24-83y (N = 81)	−0.000 (0.003) 0.010 (0.007) 0.001 (0.003)	0.94 0.13 0.64
**Parotid glands**	19 – 71 y (N = 13)	0.000 (0.003) 0.003 (0.005) -0.001 (0.005)	0.93 0.64 0.86

Betas (standard errors) and p-value are provided per probe. Values for three independent PABPN1 probes shown for datasets from Kidney cortex, Kidney medulla, Rectus *Abdominis* and Parotid glands. *P-values* are adjusted for gender. Significant changes are highlighted in bold. N indicates number of samples. Age is indicates in years (y).

### PABPN1 down-regulation (-DR) induces myogenic defects and muscle cell aging

**Figure 6:**
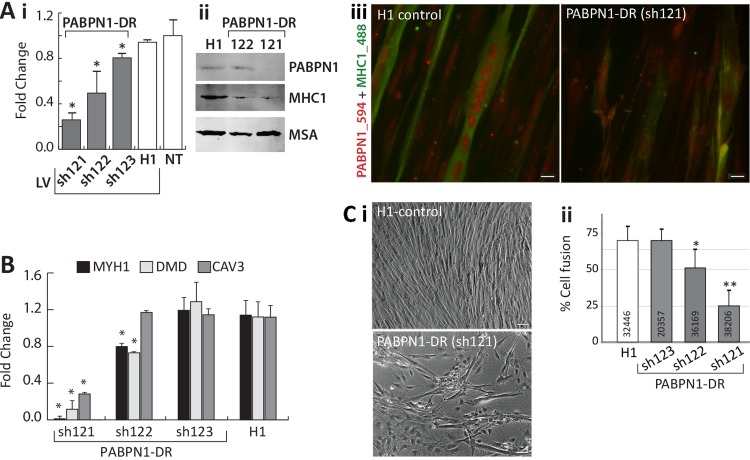
PABPN1-DR in human myotubes causes myogenic defects Human myotubes were transduced with shRNA specific to PABPN1 (sh121, sh122, or sh123) or H1 empty vector. Non-transduced (NT) cells were used as controls. (**A**) **i** Bar-chart shows PABPN1 mRNA expression in stably-transduced myoblasts. Fold change was normalized to *GapDH* housekeeping gene and to a non-transduced culture. Averages are of 6 biological replicates. **ii** Western blot analysis of PABPN1, MHC1 and muscle actin (MSA), as a loading control in sh121, sh122 or H1 myotube cultures. **iii** Immunofluorescence of PABPN1 (labelled with Alexa-594) and MHC1 (labelled with Alexa-488) in sh121 or H1 myotube cultures. Scale bar 10 μm. (**B**) Bar chart shows fold change of *MHC1*, *DMD*, and *CAV3* in 121-, 122-, 123-, and H1- myoblast cultures. Fold change was normalized to *GapDH* and to a non-transduced culture. Averages are of 3 biological replicates. Significant down-regulation (*P*<0.05) is indicated with asterisks. (**C) i-** images of H1 controls and the PABPN1 down regulation sh121 fused cultures. Scale bar is 50 μm. **ii-** Chart bar shows the percentage of nuclei in fused myotubes that express MHC1 (cell fusion) in H1 sh123 sh122 or sh121 myotube cultures. Averages and SD are from six replicates and the number of nuclei that were quantified per sample is indicated within each bar. Significant effect in PABPN1-DR cultures from control cultures (*P*<0.05 or *P*<0.005) is indicated with one or two asterisks, respectively.

Knockdown of PABPN1 in mouse muscle cells causes myogenic defects [18]. Since *in vivo* we found a decrease in *PABPN1* expression with age in both normal aging and in OPMD, we studied whether at with a moderate down-regulation of PABPN1 myogenic defects can also be observed. Three PABPN1 shRNA clones were selected for functional studies in immortalized human myoblast cultures using the lentivirus expression system for efficient delivery. Stable cultures were generated and down-regulation was evaluated with RT-qPCR and western blot analyses. Compared with controls (H1 empty vector and non- transduced cells), the three PABPN1 shRNA clones, 121, 122 and 123, led to a 70%, 40% and 20% decrease in *PABPN1* expression, respectively (Figure 6Ai). This decrease was also reflected in the residual protein levels (Figure 6Aii). Immunofluorescence microscopy with antibody to PABPN1 revealed a decrease in the accumulation of nuclear PABPN1 in the sh121-transduced cells (Figure 6Aiii).

Next, we investigated whether PABPN1-DR specifically affect expression levels of myogenic genes. We selected *MYH1*, *DMD* and *CAV3* for RT-qPCR analysis, as their expression levels significantly decreased in both OPMD and elderly. The strongest decrease in expression of all three genes was found in the sh121 cultures with highest PABPN1-DR (Figure 6B). Only a moderate decrease across all three genes was found in the sh122 culture, and expression levels did not differ from controls in the sh123 culture (Figure 6B). The decrease in *MHY1* on mRNA level was confirmed on the protein level (Figure 6A). This suggests that levels of PABPN1-DR can induce a decrease in cell fusion. An apparent decrease in cell fusion was found in the strong PABPN1-DR, sh121, cultures (Figure 6Ci). We then determined rate of cell fusion across PABPN1-DR fused cultures index using a semi-automated and robust imaging procedure, where the percentage of nuclei within cells expressing MHC1 was quantified. The rate of cell fusion was not affected in the mild PABPN1 down-regulated culture, sh123, but in sh122 or sh121 cultures cell fusion significantly reduced (Figure 6Cii). Together the results here reveal that a decrease in PABPN1 expression induces myogenic defects that are proportional to levels of PABPN1-down-regulation.

**Figure 7:**
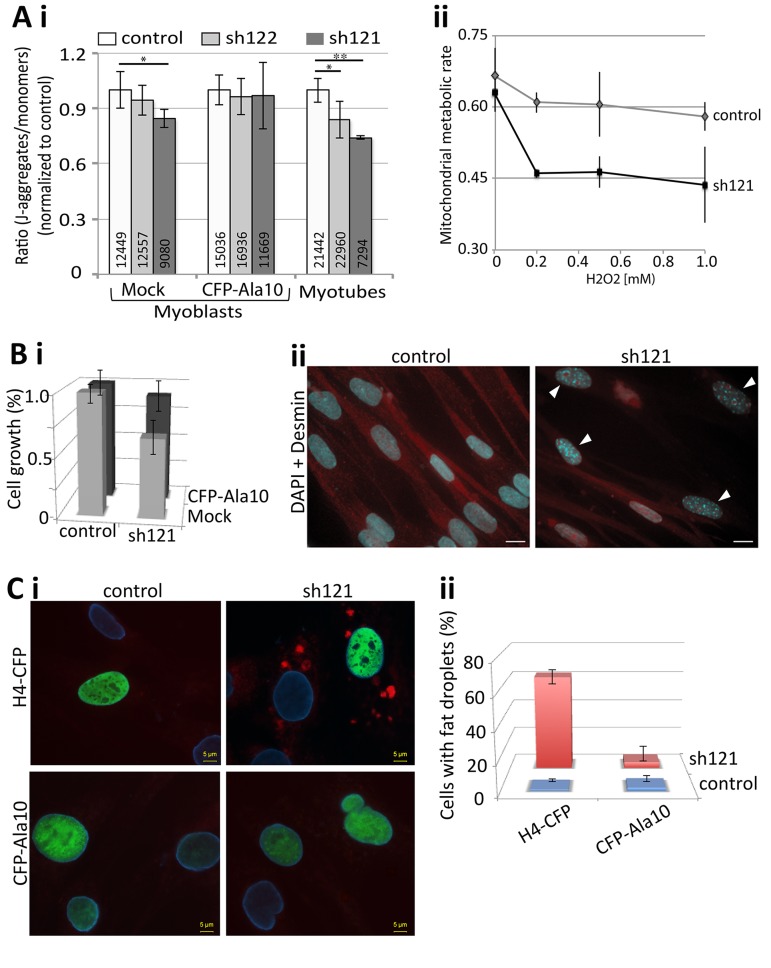
PABPN1-DR induces cellular aging in muscle cells (**A**) PABPN1-down regulation induces a decrease in mitochondrial metabolic rate. (**i**) Bar chart shows the ratio of J-aggregates to monomers in JC-1-labeled myoblasts or myotube cultures. Cell cultures are of control (7304) or PABPN1-DR (sh122 and sh121) cultures, before (mock) or after transducion with CFP-PABPN1 lentivirus particles. Averages and SD are from six replicates, and the number of cells per sample is indicated within each bar. Significant effect in PABPN1-DR from control cultures (*P*<0.05 or *P*<0.005) is indicated with one or two asterisks, respectively. (**ii**) Plot shows the ratio of J-aggregates to monomers in JC-1-labeled cultures after 1 hour H2O2, in concentrations as indicated in the chart. Averages and SD are from three replicates. (**B**) A decrease in cell growth is caused by PABPN1-down regulation. (**i**) Bar chart shows cell growth (24 hours) in control (7304) and PABPN1-DR (sh121) cultures, before (mock) or after transduction with CFP-PABPN1 lentivirus particles. Averages and SD are from three replicates. (**ii**) Heterochromatic foci are formed in PABPN1-DR cells. Heterochromatic foci are visualized with DAPI (indicated with arrowheads), cells are visualized with Desmin. Scale bar is 20 μm.

Aging cells undergo substantial cellular changes [29]. To investigate whether a decrease in PABPN1 expression induces cellular characteristics of aging cells, we applied four different cellular assays. Reduced mitochondrial metabolic rate is often found in aged skeletal muscles [30]. Using JC-1 labeling, we found a decrease in mitochondrial metabolic rate in both myoblast and myotube cultures with PABPN1 down-regulation compared with control cultures (Figure 7Ai). The effect of PABPN1-DR on the mitochondrial metabolic rate was more pronounced and more significant in myotube cultures compared with myoblasts cultures (Figure 7Ai). Importantly, a decrease in mitochondrial metabolic rate was restored in PABPN1-DR cultures after transduction with CFP-PABPN1 lentivirus particles (Figure 7Ai). Noticeably, the decrease in metabolic rate, levels of myogenic genes and fusion index correlated with levels of PABPN1-DR. As levels of mitochondrial metabolic rate are decreased due to aging-regulated increase in oxidative stress [31], next we investigated whether PABPN1 affect cell sensitivity to oxidative stress. Muscle cell cultures were treated with H_2_O_2_ and mitochondrial metabolic rate was determined after JC-1 labeling. PABPN1-DR muscle cells are 1.5-fold more sensitive to H_2_O_2_ treatment compared with control cells (Figure 7Aii). In addition, Levels of PABPN1 also affect rates of cell growth. PABPN1-DR cells have a slower growth rate compared with control, and restored after transduction with CFP-PABPN1 lentivirus particles (Figure 7Bi). In PABPN1-DR cells heterochromatic foci, which mark senescent cell [32], were also detected (Figure 7Bii). Last, we also found that PABPN1-DR induces formation of fat droplets in muscle cell cultures (Figure 7C). Importantly, restoring PABPN1 levels with CFP-PABPN1 decreased the proportion of cells with fat droplets (Figure 7Ci). Together, these studies indicate that reduced levels of PABPN1 induce muscle cell aging.

## DISSCUSION

Protein aggregation is a hallmark of a spectrum of late onset neurodegenerative disorders. A pathogenic or toxic role for aggregated proteins in general, and hence the functional relevance of expPABPN1 aggregates, to disease pathogenesis is under debate [4, 33] Since PABPN1 aggregates deplete levels of the soluble protein [10], a pathogenic model that postulates the reduced bioavailability of PABPN1 as a consequence of PABPN1 aggregation, seems equally plausible. In support, in vitro, the function of soluble PABPN1 is concentration dependent [34], and in cell models, similar changes in PAS usage are induced by either high overexpression of expPABPN1 (thus forming PABPN1 aggregates and hence depleting the cell from soluble PABPN1) or PABPN1 down-regulation [35]. In muscle cell models with similar overexpression levels of WT or expPABPN1 the changes in PAS usage were limited arguing against a gain-of-function mechanism [36]. This suggests that the levels of soluble PABPN1 rather than a gain-of function of mutant PABPN1 affects muscle cell function. Here we found that the expression of PABPN1 is dependent on age. Here we found a natural decrease in PABPN1 mRNA levels in *VL* muscles starting from the fifth decade onwards and progresses more rapid in OPMD patients compared with controls. Depletion of PABPN1 from muscle cells causes myogenesis defects [18]. Here we show that in addition to myogenesis defects, PABPN1 down-regulation induces phenotypes of muscle cell aging, including reduced cell growth rate, heterochromatic foci formation, reduced mitochondrial metabolic rate, fat droplets formation and increased sensitivity to oxidative stress. In addition, we show that levels of myogenesis and mitochondrial metabolic rate correspond to levels of PABPN1 expression. Here we provide the first indications that an age-regulated decline in PABPN1 levels, rather than gain-of function of mutant PABPN1, may cause an increase in muscle weakness. However, it is remained to be determined whether a decrease in PABPN1 levels with age is caused by increase in atrophy, increase in central nuclei, and/or increase in fibrosis, fat and inflammation in theses muscles. Further cell-cased assays should be implied to determine the change in PABPN1 levels in different cell types. In OPMD, severe muscle weakness would be the result of depletion of soluble PABPN1 due to protein aggregation and an aging-regulated decrease in expression levels.

PABPN1 is a regulator of mRNA stability [37]. Knockdown of PABPN1 expression in cells causes a decrease in poly(A) tail length [18] and a switch from distal to proximal *polyadenylation* site (PAS) usage at 3'-UTR [35, 36]. Thus, changes in PABPN1 levels could directly affect genome-wide expression profiles. In previous studies we reported that in OPMD and models with expPABPN1 overexpression transcriptomic changes are genome-wide (Anvar et al., 2011). The expression profiles in muscles from OPMD and elderly are highly similar, despite the average age difference of 30 years, suggesting that common molecular mechanisms regulate these genome-wide transcriptional changes. The mRNA processing and the spliceosome machinery were significantly deregulated in both OPMD and elderly, implying an aging-associated impairment of mRNA processing in skeletal muscles. Alternative splicing has been previously suggested to contribute to transcriptional changes during cell senescence, due to changes in expression levels of splicing factors [38]. Alternative splicing is also thought to contribute to late-onset neuro-degenerative disorders [23]. The impact of mRNA processing to genome-wide expression levels during aging and the molecular details will in the near future likely be uncovered by next generation sequencing studies.

PABPN1 is ubiquitously expressed but symptoms in OPMD are predominately restricted to a subset of skeletal muscles. We found that in OPMD patients *PABPN1* expression decreases in affected muscles but not in blood. It is important to note that expression profiles generated from muscle biopsied reflects cell heterogeneity in the actual biopsy, and those expression profiles may not be specific for muscle cells. However, age-regulated changes in PABPN1 levels were not found in six different tissues. In brain cortex a slight, but significant decline in PABPN1 expression was found from the eight decade. In *VL* muscles the decline in PABPN1 starts from the fourth decade. This suggests that a decrease in PABPN1 levels is associated with aging-associated muscle weakness and that this process is accelerated in OPMD as judged from the trend that the levels of a substantial number of genes changes faster in OPMD muscles compared to muscles of healthy controls. Together this suggests that OPMD should be regarded as a disorder of accelerated muscle aging.

## METHODS

### Pre-symptomatic carriers, patients and controls and clinical investigation

We invited 33 patients from a cohort of 10 genetically confirmed Dutch OPMD families. All symptomatic and pre-symptomatic carriers were confirmed by genetic analysis. A standard neurological examination including MRC score was performed as described previously in part of this cohort [39]. Muscle biopsies were obtained from 22 patients and 74 healthy controls (Supplementary Table 1). All biopsies are from *VL* musclesand were collected using the Bergstrom needle procedure. The biopsies froze immediately in liquid nitrogen. Blood was taken from all patients for genetic confirmation. Asymptomatic descendants of these patients were invited to participate without disclosure of their individual histological and genetic results. Eighteen of them underwent a muscle biopsy and genetic testing. Twelve descendants who did not carry the mutation were classified as controls, whereas 6 descendants who did carry the mutation where identified as pre-symptomatic. This study was approved by the local Ethical Committee (CMO nr. 2005/189) and written informed consent was obtained from all patients. Biopsies from healthy controls were collected at Canisius-Wilhelmina Hospital, Nijmegen, The Netherlands.

### Microarray and Statistical Analyses

#### Datasets

The human and mouse OPMD microarray datasets are publicly available at GEO repository under the accession numbers GSE26605 and GSE26604, respectively and have been previously published [14, 15]. In all datasets genome-wide expression profiles of skeletal muscles from OPMD were compared to controls.

#### Data Processing

Quantile normalization was applied on the microarray raw dataset and data quality was assessed by the principal component analysis. Differentially expressed genes between two age-groups were identified by applying hierarchical linear model, using limma package in R [40] at a *P-value* ≤0.01. OPMD and pre-OPMD regulated genes were identified from age-matching control groups; and elderly regulated genes were identified from controls age 56± 5 years. Probe annotation was carried out using illuminaHumanv3BeadID (human) and illumina-Mousev1BeadID (mouse) R packages.

The meta-analysis in mouse was carried out on 104 microarray datasets from various organisms as described in [26]. The 103 datasets are listed in [26], including the A17.1 6 week dataset.

The association-weights between genes and biological concepts were mined using Anni 2.1 [24]. To compare between gene groups, the association weights were normalized to the maximum association weight. Genes with literature association <0.1 and *P*>0.05 (-log_10_>1.3) in elderly and OPMD were excluded. Cumulative Distribution Function (CDF) analysis was used to examine the distribution of association weights, and the Kolmogorov-Smirnov (KS) test was used to identify distributions that significantly differ from a theoretical distribution, threshold of *P* <10^−3^. The CDF of *Gene_i_* is defined as the proportion of genes with association weight less than or equal to that of *Gene_i_*.

A k-means clustering of affected probes was used to identify age-regulated expression trends. However, in order to optimize the clustering arrangements, average Silhouette (*S*_avg_) values are calculated for each cluster in Matlab. Clustering arrangement of partitions with *S*_avg_<0.6 were reiterated until the criteria has met. Maximum number of clusters was set to 20 to avoid overly complex clustering arrangement due to the size of the set. The cluster centroids were used to provide summarized age-dependent expression patterns for each cluster.

Mapping of affected genes to functional groups (KEGG or GO) was carried out was carried out with the *globaltest* [41] or with *DAVID* functional annotation clustering tool[42]. Gene network analysis was performed with *STRING* [43].

Statistical analyses were carried out with the SPSS software (IBM) and Matlab.

### Cell culture and Lentivirus transduction

The human 7304 immortalized myoblasts [44] were propagated in a medium containing DMEM+20% Fetal Calf Serum supplemented with an equal volume Skeletal Muscle Cell Media (PromoCell, Heidelberg, Germany) at 37 °C under 5% CO_2_. Cell fusion was carried out in a medium containing DMEM+5% Horse Serum.

The shRNA in lentivirus expression vectors 121 (TRCN0000000121), 122 (TRCN0000000122) and (TRCN0000000123) 123 were obtained from Sigma-Aldrich. An empty vector, H1, was used as a negative control. Lentivirus production and transduction was previously as described [45]. Cells were cultured with viruses (MOI ~25) overnight, followed by medium refreshing. Transduced cells were maintained in the presence of 5μg/ml puromycin, which was omitted for fusion experiments. *PABPN1* down-regulation was determined using RT-qPCR. Six independent transduction experiments were performed. Cell fusion was carried out in triplicates and cell fusion index was determined by dividing the number of nuclei in myotubes to the total number of myotubes.

The lentiviruses, CFP-PABPN1 and H4-CFP, were previously described in ([13, 45], respectively). Transduction of each virus was performed in control (7304), sh121 or sh122 cultures. Only cultures with transduction efficiency >70% were included in the studies presented here.

### RNA Analysis

All human biopsies were stored at −80 before RNA extraction. RNA extraction and RT-qPCR were performed as described in [14]. RNA extraction from culture cells was performed with NucleoSpin^®^ RNA II, MACHEREY-NAGEL, according to the manufacturer protocol and RNA was directly subjected for RT-qPCR using Cyber Green, Invitrogen. Expression levels were calculated according to the DDCT method, and were first normalized to *GUSB* housekeeping gene for human samples and GAPDH for cell culture. Fold change was calculated from controls. The statistical significance was determined with the Student's t-test. The list of primers used in this study is provided in Supplementary Table 4.

### Western blot, immunofluorescence, JC-1 and Nile-red staining and image quantification

#### Western blots

Total proteins were extracted from fused cells and subjected to western blot analysis as described in [10]. Detection of the first antibodies was conducted with the Odyssey Infrared Imaging System (LI-COR Biosciences) and suitable secondary antibodies.

#### Immunofluorescence

of fused cells was carried out on cells seeded on plastics or on collagen-coated glass plates as described in [10]. Cell cultures were fused for 7 days and after fixation and permeabilization were incubated with first and subsequently with the appropriate secondary-conjugated antibodies. The Alexa 488-, Alexa 430- or Alexa 594- conjugated secondary antibodies against primary antibodies were obtained from Molecular Probes (Invitrogen).

Primary antibodies used in this study are: mouse monoclonal anti-muscle actin (MSA) (Novocastra), (1:2000) and anti-Myosin MF20 (1:500) (Sigma-Aldrich). PABPN1 was recognized with the 3F5 llama single chain [46] antibody and rabbit-anti-VHH.

#### Nile-red staining

Nile red is a fluorescent stain for intracellular lipid droplets [47]. Cells were seeded on glass and after 24 hours were incubated with 1 μM Nile-Red (Invitrogen) for 20 minutes. Cultures were washed and mounted with citiflour containing DAPI (500 ng/ml). Nile-Red was imaged with a Cy5 filter.

#### JC-1 staining

JC-1 was used to measure the mitochondrial membrane potential [48]. 1 μM JC-1 (Invitrogen) and Hoechst (Invitrogen) for nuclear counter staining in growth media and directly added into myoblasts or 7 days fused cultures for 20 minutes. Cultures were washed and living cells were directly imaged with the ArrayScan VTI HCA (Thermo Scientific) using XF53 filter set. Nuclei were segmented with the nuclear counterstain and the ratio of red to green signal was quantified from compartments surrounding each nucleus using the Compartmental Analysis V4 BioApplication (Thermo Scientific). Plots were generated in Excel.

#### Cell fusion

Fusted cultures that were immunelabeled with MF20 and counterstained nuclei were scanned with the ArrayScan VTI HCA (Thermo Scientific) using UV to detect nuclei and FITC to detect the MF20 signal. The percentage of nuclei within MF20 objects was identified with the Colocalization V4 BioApplication (Thermo Scientific). Plots were generated in Excel.

## SUPPLEMENTAL DATA


