# Supplementary material for: A decline in PABPN1 induces progressive muscle weakness in Oculopharyngeal muscle dystrophy and in muscle aging
Source: Aging (Albany NY). 2013 Jun 16;5(6):412–26. doi: 10.18632/aging.100567 (PMC3824410; doi:10.18632/aging.100567)
Supplement: Supplementary file 1 [file aging-05-412-s001.pdf]

Supplementary Table 1 – A list of muscle biopsies of OPMD patients and controls that were used for RNA expression analyses. All expPABPN1 carriers were confirmed by sequence analysis.

				**expPABPN1 Carriers**					
**Controls**				**Pre-Symptomatic**			**Symptomatic**		
**Gender**	**Age**	**Sex**	**Age**	**Gender**	**Age**	**MRC score***	**Gender**	**Age**	**MRC score***
Female	17	Male	41	Female	37	5	Female	49	4
Male	17	Male	42	Female	37	5	Female	54	5
Female	19	Male	42	Male	38	5	Female	57	4
Female	19	Male	42	Female	39	5	Male	59	5
Male	20	Male	42	Female	39	5	Female	60	4
Female	20	Male	43	Female	41	5	Female	60	4.5
Male	22	Male	43			5	Male	66	4.5
Male	23	Female	43				Male	68	3.5
Female	25	Female	43				Female	69	4.5
Female	27	Female	44						
Male	27	Male	44						
Female	27	Male	45						
Male	28	Female	48						
Male	28	Male	49						
Male	29	Female	49						
Male	29	Male	55						
Female	31	Female	56						
Female	31	Female	56						
Male	32	Male	56						
Female	32	Male	58						
Female	32	Female	58						
Female	34	Female	60						
Female	34	Female	60						
Male	34	Female	60						
Female	35	Male	60						
Female	35	Male	61						
Female	35	Male	66						
Male	36	Female	67						
Female	36	Male	67						
Male	36	Female	67						
Female	37	Female	67						
Female	38	Male	68						
Male	39	Female	70						
Female	39	Male	70						
Male	39	Male	73						
Male	40	Male	77						
Male	40	Female	85						
Male	40	Female	87						
Female	40	Female	89						

* MRC score in left and right quadriceps was determined at the same day when biopsies were sampled. Values show an average of both sides. MRC in age-matching controls and in pre-symptomatic is 5, indicating normal muscle strength; <5 indicates clinical muscle weakness.

Supplementary Table 2 - Functional Analysis of ageing and OPMD associated transcriptional changes. Biological processes are defined and clustered according to KEGG. The proportion of deregulated genes are depicted by %D.E. Genes.

			Muscle Ageing						OPMD, Anvar et al. (2011)							
			Human, Welle et al. (2004)			Human			Human		Mouse			Drosophila		
**Metabolism**			# Genes	P-value	% D.E. Genes	# Genes	P-value	% D.E. Genes	P-value	% D.E. Genes	# Genes	P-value	% D.E. Genes	# Genes	P-value	% D.E. Genes
**Carbohydrate**	620	Pyruvate metabolism	41	1.13E-07	36.59	40	1.24E-04	37.50	-		50	6.26E-09	32.00	43	6.75E-04	25.58
	51	Fructose and mannose metabolism	35	3.02E-03	20.00	35	2.11E-04	48.57	2.28E-02	28.57	40	1.18E-10	37.50	31	9.43E-04	16.13
	562	Inositol phosphate metabolism	49	3.12E-02	16.33	47	8.22E-04	31.91	2.98E-03	17.02	63	2.37E-07	31.75	32	1.89E-03	21.88
**Energy**	190	Oxidative phosphorylation	112	1.87E-05	54.46	108	7.87E-03	28.70	1.37E-02	17.59	123	4.63E-06	32.52	97	6.17E-04	49.48
	910	Nitrogen metabolism	24	6.76E-05	25.00	24	2.79E-02	50.00	1.13E-02	20.83	23	1.23E-11	43.48	27	1.21E-03	22.22
**Lipid**	564	Glycerophospholipid metabolism	67	3.39E-03	19.40	64	5.03E-07	48.44	1.67E-03	18.75	67	8.17E-09	35.82	113	8.84E-04	11.50
	1030	Glycan structures - biosynthesis 1	118	8.77E-03	23.73	106	4.30E-03	37.74	3.27E-03	14.15	137	2.54E-07	31.39	49	4.87E-02	12.24
	590	Arachidonic acid metabolism	54	1.28E-02	14.81	50	2.94E-04	40.00	1.46E-02	18.00	69	3.74E-11	24.64		-	
**Nucleotide**	230	Purine metabolism	145	5.06E-04	23.45	139	5.59E-05	43.88	5.47E-03	28.78	164	3.14E-08	35.37	246	9.39E-04	21.14
	240	Pyrimidine metabolism	89	1.56E-03	20.22	86	1.14E-03	36.05	1.94E-02	22.09	104	2.05E-08	36.54	68	1.33E-03	14.71
**Amino Acid**	260	Glycine, serine and threonine metabolism	42	3.58E-06	23.81	39	1.28E-03	30.77	4.38E-02	20.51	46	5.92E-08	23.91	34	6.36E-04	17.65
	251	Glutamate metabolism	24	1.06E-04	29.17	23	4.15E-03	47.83	4.15E-02	17.39	27	4.65E-09	40.74	26	1.25E-03	34.62
	271	Methionine metabolism	21	1.44E-02	28.57	20	1.19E-02	40.00	4.27E-02	25.00	22	4.71E-08	59.09		-	
**Glycan Biosynthesis**	1032	Glycan structures - degradation	31	2.53E-02	22.58	29	1.69E-02	37.93	3.01E-03	24.14	31	2.61E-07	29.03		-	
**Cofactors and Vitamins**	760	Nicotinate and nicotinamide metabolism	23	1.74E-06	34.78	23	2.19E-04	56.52	6.21E-03	34.78	25	1.05E-06	32.00	8	2.74E-03	12.50

**Genetic Information Processing**																
**Transcription**	3020	RNA polymerase	25	9.28E-04	28.00	22	3.94E-04	36.36	2.11E-02	27.27	31	1.21E-06	32.26	19	1.14E-03	21.05
**Translation**	970	Aminoacyl-tRNA biosynthesis	41	1.19E-03	24.39		-		1.23E-02	28.95	53	2.34E-06	35.85	26	2.92E-03	23.08
	3010	Ribosome		-		83	5.86E-03	37.35	-		67	1.73E-08	37.31	78	6.99E-04	53.85
**Folding, Sorting and Degradation**	3050	Proteasome	43	4.67E-04	32.56		-		9.27E-03	34.09	52	3.72E-06	30.77	42	7.71E-04	57.14
	4120	Ubiquitin mediated proteolysis	131	1.64E-03	27.48	126	5.41E-06	45.24	1.52E-03	30.16	177	3.61E-10	38.98	80	8.66E-04	21.25
	4130	SNARE interactions in vesicular transport	37	1.88E-03	27.03	38	5.83E-03	34.21	3.54E-02	26.32	36	2.86E-09	50.00	22	3.38E-02	9.09
**Replication and Repair**	3030	DNA replication	36	1.40E-03	33.33	34	1.25E-03	41.18	1.54E-02	23.53	38	2.97E-08	42.11	28	1.02E-03	14.29
	3420	Nucleotide excision repair	43	2.87E-02	18.60	41	1.56E-03	36.59	8.88E-04	24.39	43	3.36E-10	53.49	33	1.06E-03	24.24

Environmental Information Processing

**Membrane Transport**	2010	ABC transporters - General	41	8.85E-03	24.39	44	2.06E-04	43.18	4.08E-03	25.00	41	5.22E-09	39.02	3	3.81E-02	66.67
**Signal Transduction**	4330	Notch signaling pathway	46	1.12E-05	41.30		-		2.81E-03	18.60	49	2.70E-08	22.45	21	6.99E-03	19.05
	4150	mTOR signaling pathway	50	2.39E-05	42.00	49	1.92E-03	44.90	1.71E-02	28.57	64	3.78E-08	39.06	23	7.76E-04	34.78
	4370	VEGF signaling pathway	73	1.68E-03	23.29	67	2.48E-06	43.28	5.52E-03	20.90	82	8.69E-11	40.24	32	7.22E-04	31.25
	4010	MAPK signaling pathway	265	1.91E-03	24.91	253	7.50E-05	39.13	5.50E-03	18.18	283	1.49E-10	33.92		not available	
	4012	ErbB signaling pathway	87	1.97E-03	27.59	83	3.14E-06	33.73	2.33E-02	27.71	107	2.21E-09	35.51	26	1.31E-03	34.62
	4310	Wnt signaling pathway	148	4.43E-03	25.68	147	1.47E-04	39.46	7.60E-04	21.09	171	1.78E-10	35.09	64	8.77E-04	23.44
	4350	TGF-beta signaling pathway	83	7.54E-03	25.30	82	8.16E-05	39.02	9.51E-03	26.83	84	1.70E-10	38.10	29	1.36E-02	17.24
	4340	Hedgehog signaling pathway	53	1.95E-02	16.98	56	3.30E-02	26.79	3.69E-03	16.07	56	7.04E-09	21.43	18	1.15E-03	33.33
	4070	Phosphatidylinositol signaling system	76	2.16E-02	18.42	75	4.29E-03	34.67	3.44E-03	20.00	94	3.85E-10	34.04	45	2.64E-03	13.33
	4630	Jak-STAT signaling pathway	150	2.24E-02	19.33	145	3.92E-05	39.31	5.59E-03	19.31	170	7.91E-09	25.29	20	1.80E-03	35.00
	4020	Calcium signaling pathway	176	4.28E-02	21.59	169	3.05E-04	41.42	1.36E-03	18.34	220	6.19E-10	26.36	43	6.54E-04	32.56
**Signaling Molecules and Interaction**	4512	ECM-receptor interaction	84	3.93E-02	20.24	79	5.33E-03	43.04	5.01E-04	27.85	97	1.72E-05	31.96		not available	

Cellular Processes

**Transport and Catabolism**	4140	Regulation of autophagy	34	1.34E-03	26.47	32	1.95E-02	31.25	3.59E-02	18.75	39	8.13E-08	30.77	16	1.07E-03	25.00
**Cell Motility**	4810	Regulation of actin cytoskeleton	210	7.61E-03	22.38	204	9.77E-03	33.33	2.43E-02	16.67	229	2.44E-08	32.75		not available	
**Cell Growth and Death**	4110	Cell cycle	111	1.39E-04	28.83	109	4.98E-06	42.20	6.02E-03	21.10	119	3.80E-09	36.97		-	
	4115	p53 signaling pathway	68	1.39E-03	27.94	63	1.12E-06	34.92	-		75	7.69E-11	37.33	15	7.20E-04	6.67
	4210	Apoptosis	88	4.61E-03	23.86	78	5.43E-06	42.31	1.09E-02	21.79	104	1.74E-10	31.73	19	6.06E-04	36.84
**Cell Communication**	4510	Focal adhesion	199	9.03E-04	26.13	190	3.13E-04	44.21	3.38E-03	24.74	215	4.04E-08	37.21		not available	
	4520	Adherens junction	74	9.49E-04	28.38	75	1.57E-02	34.67	3.83E-03	25.33	84	1.17E-07	32.14		not available	
	4530	Tight junction	129	1.72E-03	22.48	127	1.87E-03	40.16	2.13E-02	21.26	145	2.15E-09	37.93		not available	
	4540	Gap junction	94	8.11E-03	23.40	92	1.62E-03	40.22	9.79E-04	31.52	105	2.40E-09	31.43		not available	

Organismal Systems

**Immune System**	4660	T cell receptor signaling pathway	94	5.71E-03	23.40	86	7.00E-03	45.35	3.53E-02	15.12	112	1.11E-09	27.68		not available	
	4670	Leukocyte transendothelial migration	113	1.29E-02	17.70	107	6.69E-03	45.79	-		125	1.25E-08	25.60		not available	
	4664	Fc epsilon RI signaling pathway	76	1.87E-02	17.11	73	4.12E-04	45.21	1.37E-03	17.81	86	1.21E-10	30.23		not available	
	4650	Natural killer cell mediated cytotoxicity	130	2.38E-02	17.69	121	1.49E-03	42.15	3.19E-02	11.57	138	3.86E-10	26.09		not available	
	4620	Toll-like receptor signaling pathway	101	3.70E-02	17.82	93	1.22E-02	45.16	1.62E-02	19.35	108	1.06E-10	30.56		not available	
**Endocrine System**	4910	Insulin signaling pathway	136	8.43E-04	30.88	135	3.26E-05	39.26	1.62E-03	29.63	161	2.36E-08	38.51		not available	
	4912	GnRH signaling pathway	98	1.05E-02	23.47	95	4.96E-05	40.00	7.40E-04	30.53	116	3.47E-10	32.76		not available	
	4916	Melanogenesis	99	1.74E-02	20.20	101	5.49E-03	30.69	6.92E-04	25.74	108	2.54E-10	35.19		not available	
**Nervous System**	4720	Long-term potentiation	70	9.74E-03	25.71	68	5.89E-03	36.76	2.58E-03	25.00	85	2.00E-11	36.47		not available	

Human Diseases

**Cancer**	5211	Renal cell carcinoma	69	3.72E-05	34.78	67	1.85E-05	40.30	3.06E-02	20.90	74	2.42E-09	40.54	not available
	5216	Thyroid cancer	29	1.65E-04	41.38	28	4.66E-04	42.86	1.18E-02	25.00	28	2.75E-04	35.71	not available
	5215	Prostate cancer	90	2.30E-04	26.67	84	5.94E-03	45.24	4.69E-03	29.76	103	7.82E-08	36.89	not available
	5218	Melanoma	71	5.25E-04	30.99		-		3.93E-02	19.12	75	2.88E-08	44.00	not available
	5214	Glioma	64	7.85E-04	32.81	62	1.19E-02	38.71	5.39E-03	24.19	76	2.20E-09	50.00	not available
	5220	Chronic myeloid leukemia	74	8.54E-04	36.49	70	3.40E-05	32.86	2.81E-02	22.86	87	1.43E-08	39.08	not available
	5223	Non-small cell lung cancer	54	1.16E-03	29.63	51	1.42E-02	33.33	3.35E-02	17.65	62	4.73E-09	43.55	not available
	5210	Colorectal cancer	83	1.42E-03	31.33	81	2.71E-03	38.27	1.71E-02	20.99	92	9.70E-09	42.39	not available
	5222	Small cell lung cancer	86	2.19E-03	27.91	78	2.71E-04	53.85	1.31E-02	25.64	108	3.74E-07	35.19	not available
	5213	Endometrial cancer	52	2.36E-03	26.92	50	2.30E-03	40.00	3.81E-02	24.00	60	8.46E-08	40.00	not available
	5221	Acute myeloid leukemia	57	4.54E-03	29.82	53	1.83E-05	47.17	2.99E-02	28.30	71	5.10E-07	30.99	not available
	5217	Basal cell carcinoma	53	2.66E-02	18.87	53	4.26E-03	33.96	2.31E-04	16.98	54	1.08E-06	27.78	not available
**Immune System**	5340	Primary immunodeficiency	34	1.24E-02	14.71		-		3.51E-02	12.50	36	2.02E-08	11.11	not available
**Neurodegenerative**	5010	Alzheimer's disease	158	5.04E-06	46.84	151	6.45E-05	29.80	2.57E-03	21.85	187	1.00E-09	33.16	not available
	5012	Parkinson's disease	113	1.35E-05	58.41	104	2.33E-03	27.88	2.11E-02	18.27	138	1.93E-09	35.51	not available
	5040	Huntington's disease	31	2.93E-03	32.26	28	9.57E-03	42.86	5.01E-03	35.71	38	6.79E-08	36.84	not available
	5014	Amyotrophic lateral sclerosis (ALS)	55	6.35E-03	27.27	51	7.47E-05	37.25	1.04E-02	23.53	67	5.40E-10	26.87	not available
**Metabolic**	4930	Type II diabetes mellitus	42	2.49E-03	35.71	41	7.43E-05	36.59	2.58E-02	19.51	51	1.97E-09	33.33	not available
**Infectious**	5130	Pathogenic Escherichia coli infection - EHEC	49	1.28E-03	38.78	48	4.76E-02	45.83	2.28E-02	22.92		-		not available
	5110	Vibrio cholerae infection	56	2.39E-03	25.00	57	5.07E-03	45.61	3.67E-03	28.07		-		not available
	5120	Epithelial cell signaling in Helicobacter …	65	1.16E-02	20.00	62	1.58E-05	53.23	2.53E-02	29.03		-		not available

eTable 2 -

A list of genes that exhibit accelerated expression trends in OPMD compared with skeletal muscle aging.

Symbol	Entrez IDs	Probe IDs	A	S	P
ABCA11	79963	2320403	Blue	Blue	
ACP6	51205	4670671	Blue	Blue	Blue
ADAM7	8756	1260424	Blue	Blue	
ALDH3A1	218	5560369	Yellow	Yellow	Yellow
ALDH6A1	4329	5820373	Yellow	Yellow	Yellow
AP2A1	160	4390180	Yellow	Yellow	
BHLHB5	27319	7210551	Yellow	Yellow	
C21ORF34	388815	70368	Blue	Blue	
C4ORF29	80167	5810192	Yellow	Yellow	
CDKN1A	1026	4230201	Yellow	Yellow	
CHRNA1	1134	1410017	Yellow	Yellow	Yellow
CHRNA1	1134	4120717	Yellow	Yellow	Yellow
CHRNA1	1134	4570634	Yellow	Yellow	Yellow
CREB5	9586	4290619	Yellow	Yellow	Yellow
CTDSP1	58190	7150333	Yellow	Yellow	Yellow
DHRS12	79758	3710086	Blue	Blue	Blue
DNAJB6	10049	6580189	Yellow	Yellow	
DUOX1	53905	3060528	Blue	Blue	
EEF1A1	1915	3450719	Yellow	Yellow	
EGFLAM	133584	7380092	Yellow	Yellow	
EMX1	2016	3460390	Blue	Blue	Blue
FRAS1	80144	270358	Blue	Blue	Blue
GRM2	2912	4230673	Blue	Blue	
H2AFX	3014	4120538	Yellow	Yellow	
HPS4	89781	3170504	Blue	Blue	
IARS	3376	6420397	Blue	Blue	
IGF2BP3	10643	1770243	Yellow	Yellow	Yellow
JARID2	3720	2320286	Blue	Blue	Blue
LMOD1	25802	6760246	Blue	Blue	Blue
LOC100131174	100131174	4670482	Blue	Blue	
LOC400793	400793	5090142	Yellow	Yellow	
LOC402251	402251	3440670	Yellow	Yellow	
LOC642795	642795	6100068	Yellow	Yellow	
LOC644284	644284	2490451	Yellow	Yellow	
LOC649260	649260	770348	Yellow	Yellow	
LRTM2	654429	5890243	Yellow	Yellow	Yellow
MAP2K3	5606	4200544	Blue	Blue	
METTL10	399818	6370075	Blue	Blue	
MUTYH	4595	5670037	Blue	Blue	
NEK3	4752	1190504	Blue	Blue	
NFYA	4800	6180072	Yellow	Yellow	
OSBP	5007	5550296	Blue	Blue	
PDDC1	347862	3420730	Blue	Blue	Blue
PHF19	26147	5550402	Blue	Blue	
POU5F1	5460	5550577	Blue	Blue	
PRODH	5625	5860689	Yellow	Yellow	
PRRX1	5396	1440601	Yellow	Yellow	Yellow
RAPGEFL1	51195	1940072	Blue	Blue	Blue
RCC1	1104	2570292	Yellow	Yellow	
RDH5	5959	110326	Blue	Blue	
RFPL3S	10737	1240731	Blue	Blue	
ROPN1B	152015	730521	Yellow	Yellow	
SF3A3	10946	1230747	Blue	Blue	
SH2B3	10019	6560301	Yellow	Yellow	
SLC1A4	6509	6350392	Blue	Blue	Blue
SLC36A2	153201	6180711	Blue	Blue	
SLC38A10	124565	6590333	Yellow	Yellow	
SNHG11	128439	4560332	Yellow	Yellow	Yellow
SPCS2	9789	7040068	Blue	Blue	
STX11	8676	60470	Yellow	Yellow	
STX5	6811	6450470	Yellow	Yellow	
TIAM2	26230	7050612	Yellow	Yellow	Yellow
TMEM55B	90809	5220152	Blue	Blue	Blue
TRIM38	10475	3930377	Yellow	Yellow	
TSC1	7248	4040373	Blue	Blue	
TSPY1	64591	4250097	Blue	Blue	
UBQLN4	56893	2120215	Yellow	Yellow	Yellow
UCP2	7351	6580059	Yellow	Yellow	Yellow
UNC13B	10497	5820121	Blue	Blue	Blue
UPB1	51733	3830735	Blue	Blue	Blue
VAMP1	6843	2350541	Blue	Blue	
WDR20	91833	3130494	Blue	Blue	
ZCCHC11	23318	4260093	Blue	Blue	Blue
ZNF679	168417	3940431	Yellow	Yellow	

A: Aging
S: Symptomatic
P: Pre-symptomatic

Yellow: Up-regulation
Blue: Down-regulation

Supplementary Table 4 – A primer list for qPCR.

Gene	FW Primer	RV Primer
GUSB	5’ CTCATTTGGAATTTTGCCGATT	5’ CCGAGTGAAGATCCCCTTTTTA
GapDH	5’ CAACGAATTTGGCTACAGCA	5’ AGGGGTCTACATGGCAACTG
PABPN1	5’ CGTTGGCAATGTGGACTATG	5’ ACACGGTTGACTGAACCACA
MYH1	5’ TGGACAAACTGCAAGCAAAG	5’ GACCTGGGACTCAGCAATGT
CAV3	5’ CTGTTGCCTGAGCACAAAAA	5’ GTTAGCCAAAGGGGAGGTTC
